# Supplementary material for: The interactome of intact mitochondria by cross-linking mass spectrometry provides evidence for coexisting respiratory supercomplexes
Source: Mol Cell Proteomics. 2017 Dec 8;17(2):216–32. doi: 10.1074/mcp.RA117.000470 (PMC5795388; doi:10.1074/mcp.RA117.000470)
Supplement: Supplemental Data [file supp_RA117.000470_133922_0_supp_23978_fzffwf.zip › spectra_annotation/mito_DR_spectra_annotation/92-1-8-1-2-1.pdf]

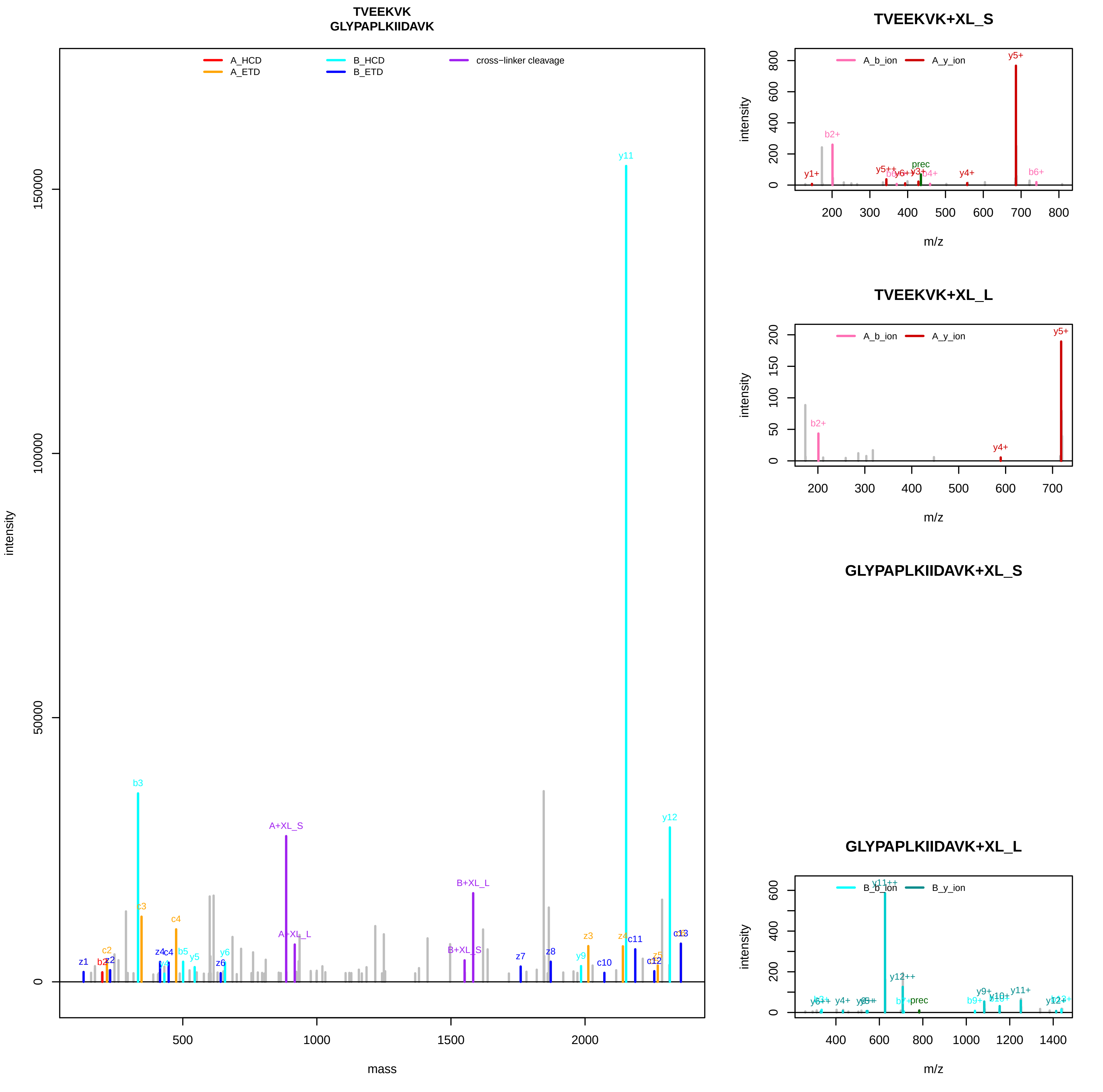
TVEEKVK
GLYPAPLKIIDAVK
A_HCD
A_ETD
B_HCD
B_ETD
cross−linker cleavage
intensity
mass
0
50000
100000
150000
500
1000
1500
2000
y11
b3
y12
A+XL_S
A+XL_L
B+XL_L
B+XL_S
c3
c4
c2
z1
z2
z4
c4
b5
y5
z6
y6
z7
z8
z3
y9
c10
c11
c12
c13
TVEEKVK+XL_S
A_b_ion
A_y_ion
y5+
b2+
y1+
prec
y4+
b6+
m/z
TVEEKVK+XL_L
y4+
GLYPAPLKIIDAVK+XL_S
GLYPAPLKIIDAVK+XL_L
B_b_ion
B_y_ion
y11++
y12++
y4+
y9+
y10+
y11+
prec
